# Phloretin Modulates STING Ubiquitination Degradation to Restrain Dendritic Cell Overactivation and Alleviate Sjögren’s Syndrome

**DOI:** 10.3390/ph19091398

**Published:** 2026-09-04

**Authors:** Tianle Zhan, Haoran Chen, Jian Yao, Chuangqi Yu

**Affiliations:** 1 Department of Oral Surgery, Shanghai Ninth People’s Hospital, School of Medicine, College of Stomatology, Shanghai Jiao Tong University, No. 639, Zhizaoju Rd, Shanghai 200011, China; ntztl@hotmail.com; 2 National Center for Stomatology, National Clinical Research Center for Oral Diseases, Shanghai Key Laboratory of Stomatology, Shanghai Research Institute of Stomatology, No. 639, Zhizaoju Rd, Shanghai 200011, China; 3 Departmentof Interventional Therapy, Shanghai Ninth People’s Hospital, School of Medicine, College of Stomatology, Shanghai Jiao Tong University, No. 639, Zhizaoju Rd, Shanghai 200011, China; 4 Department of Histology and Embryology, Medical School, Nantong University, No. 19 Qixiu Road, Nantong 226007, China

**Keywords:** Sjögren’s syndrome, phloretin, ubiquitination degradation, dendritic cells, STING, poly I:C

## Abstract

**Background/Objectives**: Sjögren’s Syndrome (SS) is an autoimmune disorder with impaired exocrine gland function. Its pathogenesis remains elusive, but aberrant innate/adaptive immunity and dysregulated interferon signaling are core features. Current SS research lacks exploration of innate immune mechanisms and therapeutic targets. Phloretin, a natural dihydrochalcone derivative, inhibits excessive innate immune activation, yet its role and mechanism in SS remain unreported. **Methods**: This study aimed to explore the therapeutic efficacy of phloretin against Sjögren’s syndrome (SS) and elucidate the regulatory mechanism underlying its effects on dendritic cells (DCs) and the Stimulator of Interferon Genes (STING)-driven innate immune cascade. Clinical specimens were utilized to analyze DC infiltration and STING expression in the labial glands of SS patients. In vivo experiments were conducted on NOD/Ltj SS mice with phloretin treatment, using hydroxychloroquine as a positive control. For in vitro studies, DCs were stimulated with poly I:C to verify the functional effects of phloretin and its regulatory role in the STING/TANK-binding kinase 1 (TBK1)/Interferon Regulatory Factor 3 (IRF3) signaling. Salivary secretion, glandular inflammatory responses, and systemic immune overactivation were evaluated in SS mice. In poly I:C-stimulated DCs, cell activation, migration, and co-stimulatory molecule expression were measured. The regulatory effects of phloretin on the STING signaling cascade and STING ubiquitin-mediated degradation were examined by Western blotting. Phloretin, initially identified as a candidate compound via virtual screening, was subjected to thermal shift assay (CETSA), drug affinity responsive target stability (DARTS) and molecular dynamics simulation (MD) for direct binding verification with STING. The STING overexpression rescue experiment further corroborates that phloretin modulates innate immune responses in a STING-dependent manner. **Results**: Clinical results revealed obvious infiltration of CD11c^+^STING^+^ DCs and CD11c^+^p-TBK1^+^ DCs in labial gland lesions of SS patients. In NOD/Ltj SS mice, phloretin treatment recovered salivary secretion, relieved glandular inflammatory injury, and restrained excessive activation of innate and adaptive immune responses, showing comparable therapeutic effects compared with hydroxychloroquine. In vitro experiments confirmed that phloretin could alleviate poly I:C-induced abnormal activation and migration of DCs, as well as reduce the expression of co-stimulatory molecules. Mechanistically, phloretin was found to interfere with the STING signaling cascade, alter the ubiquitination level of STING protein, and further inhibit the abnormal activation of DCs, which may contribute to its protective effects against Sjögren’s syndrome. Notably, results from MD, CETSA and DARTS collectively validate the interaction between phloretin and STING protein, providing solid molecular-level evidence for its regulatory effect on STING signaling transduction. **Conclusions**: DCs and STING-driven innate immunity critically contribute to SS progression. Phloretin effectively ameliorates SS-related immune disorders by restraining DC overactivation and STING signaling, supporting it as a promising candidate agent for SS treatment.

## 1. Introduction

Sjögren’s syndrome (SS) is an autoimmune disease of unknown etiology, characterized by immune cell infiltration of exocrine glands and progressive functional damage [1,2,3,4]. It is the second most common autoimmune disease after rheumatoid arthritis, with 35% of patients complicated by rheumatoid arthritis [5,6,7,8].

Uncontrolled SS increases the risk of lymphoma 4–16 times compared with healthy people, highlighting the importance of early treatment. Currently, clinical drugs such as glucocorticoids and methotrexate have significant side effects with long-term use, so there is an urgent need for effective therapeutic drugs with low cytotoxicity [6,7,8,9,10]. Early work [5,6,7,8] identified T and B cells as the primary drivers of lymphocytic infiltration in Sjögren’s syndrome, owing to their prominent accumulation in glandular lesions. Nonetheless, the contribution of upstream innate immune cells remains poorly explored. Emerging data [11,12,13] underscores the vital function of innate immunity in disease pathogenesis. Modulating upstream innate immune pathways may thus provide a promising strategy for therapeutic intervention. Dendritic cells (DCs) may contribute to SS pathogenesis. They recognize and present self-nucleotides, activate and polarize T cells into Th1 and Th17 cells, induce local immune responses, and promote pathological progression. STING activation can stimulate type I interferon production, enhance immune responses, DC maturation and T cell-mediated adaptive immunity [11,12,13,14,15,16,17,18]. Modulating STING signaling has therapeutic potential in various autoimmune diseases, but its role in SS remains unclear.

Phloretin is a flavonoid compound with inherent anti-inflammatory and immunomodulatory properties, and accumulating high-quality studies [19,20,21] have demonstrated that flavonoids can target STING to ameliorate autoinflammatory and autoimmune disorders. As a natural product-derived agent, phloretin has exerted favorable therapeutic effects in multiple disease animal models [19,20,21]. While previous studies [22,23] have primarily focused on STING activation within salivary gland epithelial cells, which can be challenging to target effectively, this study investigates STING-mediated immune dysfunction specifically in dendritic cells. By exploring this less-examined aspect of Sjögren’s syndrome (SS) pathogenesis, our strategy aims to offer alternative avenues for improved drug targeting, which may help minimize potential adverse side effects. The present study revealed that phloretin may suppress DC activation and block STING/TBK1/IRF3 signaling transduction via modulating STING signaling in DCs, and further downregulate T cell-dominated adaptive immune responses, which provides a novel potential strategy for the clinical intervention of SS.

## 2. Results

### 2.1. Clinical Sample Analysis of SS

**Figure 1:**
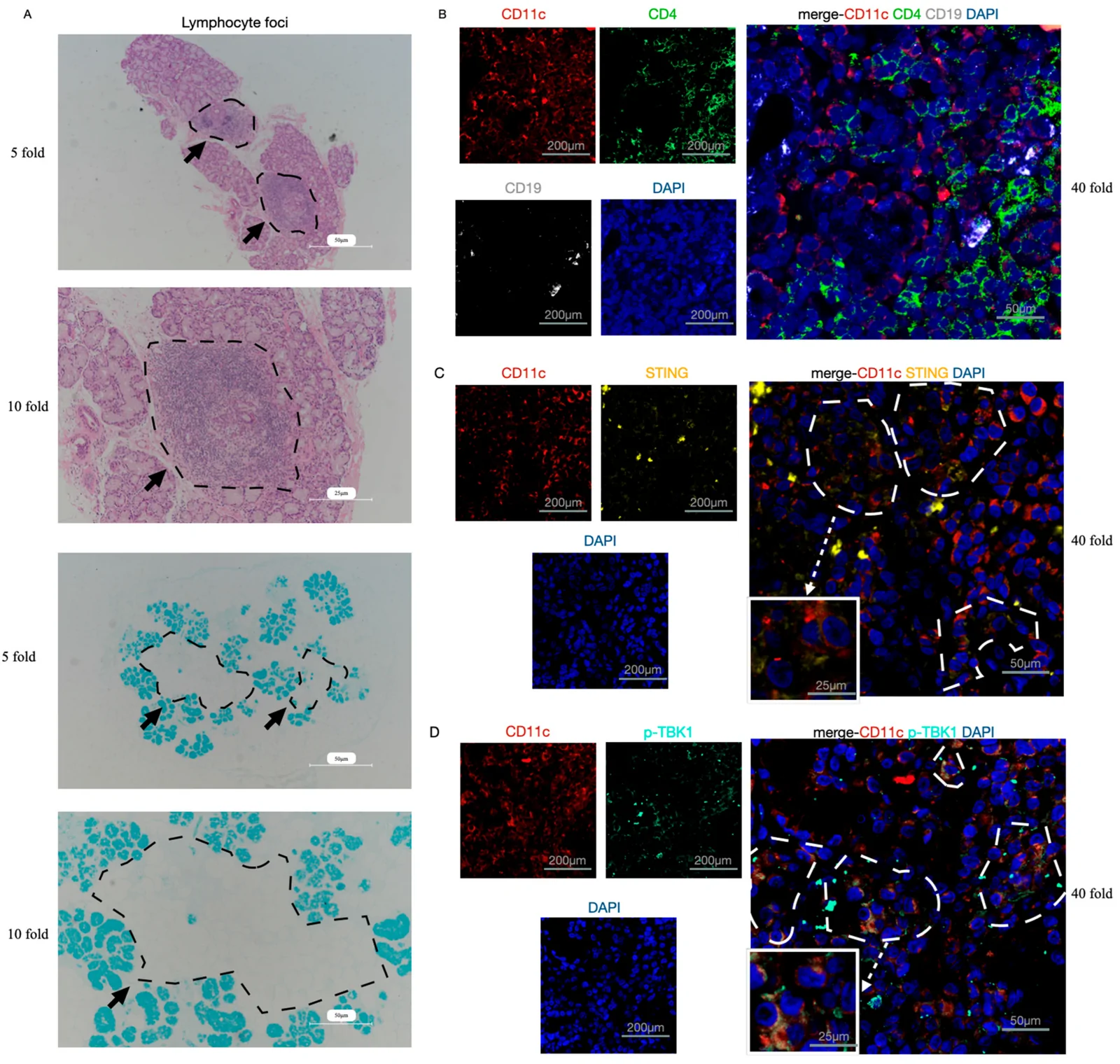
Study on labial gland samples from donors. (**A**) HE staining shows lymphocytic infiltration foci in donor labial glands. Arrows indicate lymphocytic foci; Alcian blue staining reveals regions with impaired secretory function of labial glands (magnification: 5× or 10×). Arrows indicate vacuolar regions. (**B**) Multiplex immunohistochemical analysis of donor labial glands indicates infiltration of CD4/CD19/CD11c-positive cells (magnification: 40×). (**C**) Multiplex immunohistochemical analysis of donor labial glands shows infiltration of CD11c- and STING-positive cells (magnification: 40×). Dashed circles denote regions of fluorescence colocalization. (**D**) Multiplex immunohistochemical analysis of donor labial glands demonstrates infiltration of CD11c and p-TBK1-positive cells (magnification: 40×). Dashed circles denote regions of fluorescence colocalization.

To characterize the transcriptional expression profile of the STING in primary Sjögren’s syndrome (pSS), we first analyzed three independent datasets retrieved from the Gene Expression Omnibus (GEO) database (Figure S1). These datasets included peripheral blood mononuclear cells (PBMCs) and pathologically stratified minor salivary gland (MSG) tissues collected from SS patients and healthy control subjects. Transcriptomic data revealed no statistically significant difference in STING expression in PBMCs between SS patients and healthy individuals in the GSE66795 cohort, indicating that circulating peripheral immune cells do not undergo transcriptional alterations of STING at the bulk transcriptome level. In contrast, the expression level of STING in salivary gland tissues was strongly correlated with disease progression and the severity of pathological lesions. In the disease-stage stratified GSE23117 dataset, statistically significant upregulation of STING mRNA was only observed in lesions from advanced-stage SS patients. Although a slight upward trend in STING expression was also detected in early- and moderate-stage lesions, these changes did not reach statistical significance. This finding implies that STING overexpression tends to serve as a molecular pathological hallmark of SS at the advanced or severe stage of disease progression. Further subgroup analysis based on pathological phenotypes using the GSE272410 dataset demonstrated markedly elevated transcription of STING in salivary gland tissues with tertiary lymphoid structure (TLS) lesions. Moreover, STING expression increased in a stepwise manner alongside aggravated focal lymphocytic infiltration, reaching the highest abundance in lesions with germinal center formation. Collectively, analyses of public transcriptomic datasets confirm that upregulated STING expression represents a salivary gland-specific molecular event in SS, which is closely linked to local inflammatory infiltration, tertiary lymphoid structure formation, and overall disease severity. Building on the above bioinformatic findings, we further verified the pathological expression pattern of STING protein and the activation status of the STING/TBK1 signaling pathway using clinical labial salivary gland biopsies from SS patients. This section aimed to delineate the tissue distribution of CD11c^+^ dendritic cells and their spatial correlation with local lymphocytic inflammatory foci, as well as to verify the in situ aberrant activation of the STING/TBK1 signaling axis within lesional tissues. Hematoxylin and eosin (HE) staining was performed to evaluate the typical inflammatory pathological manifestations in SS labial glands. According to the gold standard for SS pathological diagnosis, a lymphocytic focus is defined as an aggregate of no fewer than 50 lymphocytes within a 4 mm^2^ glandular area. The number and scope of lymphocytic foci directly reflect the hyperactive autoimmune inflammatory status and disease severity of individual patients. In addition, Alcian blue staining was applied to assess glandular secretory function. Normal salivary gland tissues displayed uniform pale-blue staining, whereas weakened or negative staining was detected in inflammatory damaged areas and lymphocyte-infiltrated regions, suggesting impaired mucin secretion and glandular dysfunction within SS lesions (Figure 1A). Given that the STING/TBK1 innate immune signaling pathway contributes to the pathogenesis of multiple autoimmune disorders, we further explored the in situ expression and activation of STING-related signaling molecules in SS salivary gland lesions. Inflammatory foci were first identified via HE staining, and markers for CD4^+^ T cells and CD19^+^ B cells were subsequently used to locate lymphocytic infiltration sites. Immunofluorescence combined with laser confocal microscopy showed that CD11c^+^ dendritic cells were predominantly accumulated around inflammatory foci dominated by CD4^+^ T cells and CD19^+^ B cells (Figure 1B). Notably, clear co-localization of STING and phosphorylated TBK1 was detected in CD11c^+^ dendritic cells residing within salivary gland lesions (Figure 1C,D). Combined with the supporting evidence from GEO transcriptomic data, these clinical specimen results further illustrate that aberrant activation of the STING/TBK1 signaling pathway in lesion-resident dendritic cells is deeply involved in local inflammatory responses and pathological deterioration during Sjögren’s syndrome progression.

### 2.2. Simulation-Assisted Screening of Phloretin Interacting with STING and Exploratory In Vivo Pharmacological Studies

**Figure 2:**
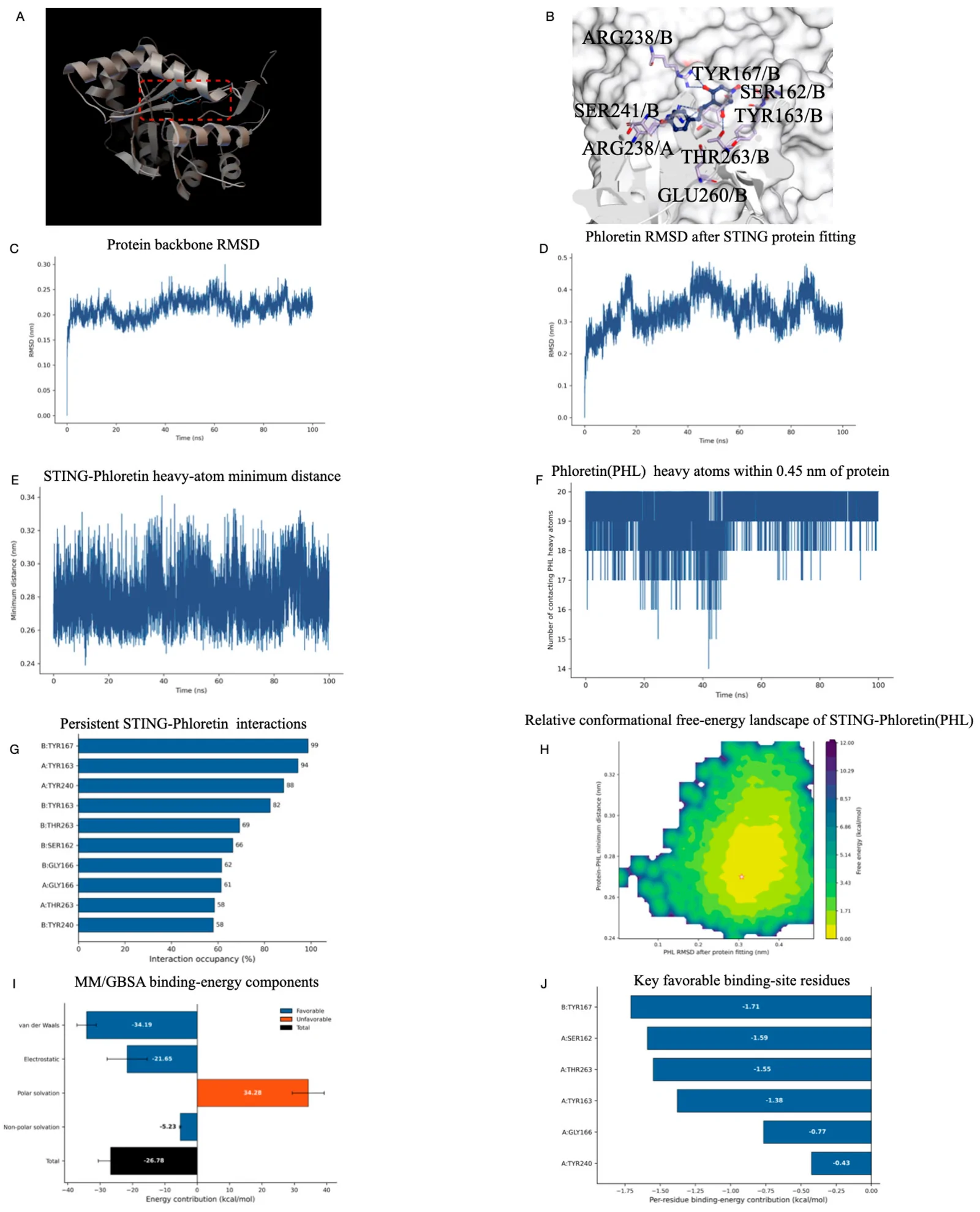
Molecular docking and 100 ns molecular dynamics (MD) simulation analyses of the binding interaction between STING protein and phloretin. (**A**) Overall three−dimensional structure of STING with the phloretin binding pocket highlighted by the red dashed box. (**B**) Detailed binding mode of phloretin within the STING active pocket; key interacting amino acid residues are labeled, showing hydrogen bonds and hydrophobic interactions between the ligand and protein. (**C**) Root-mean-square deviation (RMSD) curve of the STING protein backbone throughout the 100 ns MD simulation, reflecting the overall structural stability of the receptor protein. (**D**) RMSD curve of bound phloretin after fitting to the STING protein, indicating the conformational stability of the small molecule ligand in the binding cavity. (**E**) Time-dependent minimum heavy-atom distance between STING protein and phloretin during simulation, verifying sustained close contact between the receptor and ligand. (**F**) Number of heavy atoms of phloretin kept within 0.45 nm from the STING protein across the entire simulation trajectory, demonstrating persistent embedding of the ligand in the binding pocket. (**G**) Bar plot showing the occupancy percentage of persistent residue-level interactions between STING and phloretin during MD simulation. (**H**) Relative conformational free energy landscape of the STING-phloretin complex projected by protein RMSD and ligand RMSD, with the lowest free-energy stable conformation marked by the pink star (**I**) MM/GBSA decomposition analysis of binding free energy components for the STING-phloretin complex, including van der Waals, electrostatic, polar solvation, non-polar solvation contributions and total binding free energy. (**J**) Per-residue decomposition of favorable binding free energy for key amino acid residues at the phloretin binding site on STING.

Based on the literature review [24,25,26], preliminary pharmacological molecular docking screening and 100 ns all-atom molecular dynamics (MD) simulations (Figure 2), we propose that phloretin may serve as a potential agent regulating STING in Sjögren’s syndrome. Molecular docking results (AutoDock Vina v1.1.2 optimal docking score: −8.517 kcal/mol) revealed that phloretin could stably insert into the intracellular ligand-binding pocket of STING (Figure 2A), and anchor at the active center via non-covalent interactions including hydrogen bonds and hydrophobic interactions with multiple key amino acid residues such as SER261, GLU260, ARG238 and TYR167 (Figure 2B). Stability analysis of the 100 ns MD trajectories demonstrated that the root-mean-square deviation (RMSD) of the STING protein backbone fluctuated slightly in the early simulation stage and then rapidly plateaued, remaining within the range of 0.15–0.25 nm throughout the simulation (Figure 2C). The RMSD of bound phloretin ligand also converged quickly with minor oscillations (Figure 2D), verifying favorable conformational stability for both the protein receptor and small-molecule ligand. The minimum heavy-atom distance between the protein and ligand was steadily maintained at 0.24–0.34 nm during the whole simulation (Figure 2E), and a large number of heavy atoms of phloretin persisted within the 0.45 nm interaction radius of the protein (Figure 2F), directly indicating that phloretin did not dissociate from the binding pocket over the simulation process and formed a stable complex with STING. Statistics on persistent interactions extracted from trajectories showed that multiple residues including TYR167, TYR163, THR263 and SER162 exhibited interaction occupancy exceeding 60%, which were identified as core amino acid sites sustaining ligand binding (Figure 2G). The free energy potential of mean force (PMF) landscape presented a single dominant conformation with the lowest energy minimum, reflecting robust conformational convergence of the system (Figure 2H). MM/GBSA free energy decomposition analysis yielded a total binding free energy of −26.78 kcal/mol for the STING–phloretin complex. Van der Waals forces (−34.19 kcal/mol) and electrostatic interactions (−21.65 kcal/mol) served as the major favorable driving forces, whereas polar solvation energy contributed unfavorably to binding. Residue-level energy decomposition further pinpointed TYR167, SER162, THR263 and TYR163 as the top critical residues contributing to binding affinity (Figure 2I,J). In this exploratory pharmacological studies, Sjögren’s syndrome model mice were used with negative control group established to evaluate the therapeutic effects of phloretin. Based on previous research of our group, submandibular gland lymphocytic infiltration spontaneously occurs in 8-week-old NOD/Ltj mice. Therefore, intragastric administration was performed continuously for 8 weeks starting from the age of 8 weeks. Mice in the low- and high- dose phloretin groups were treated with 25 mg/kg and 75 mg/kg phloretin, respectively. The negative control group received 0.5% sodium carboxymethyl cellulose solution, and hydroxychloroquine (50 mg/kg) was set as the positive control. As a first line clinical drug for Sjögren’s syndrome, hydroxychloroquine [27,28], similar to phloretin, regulates innate immunity, making it suitable for this controlled study.

**Figure 3:**
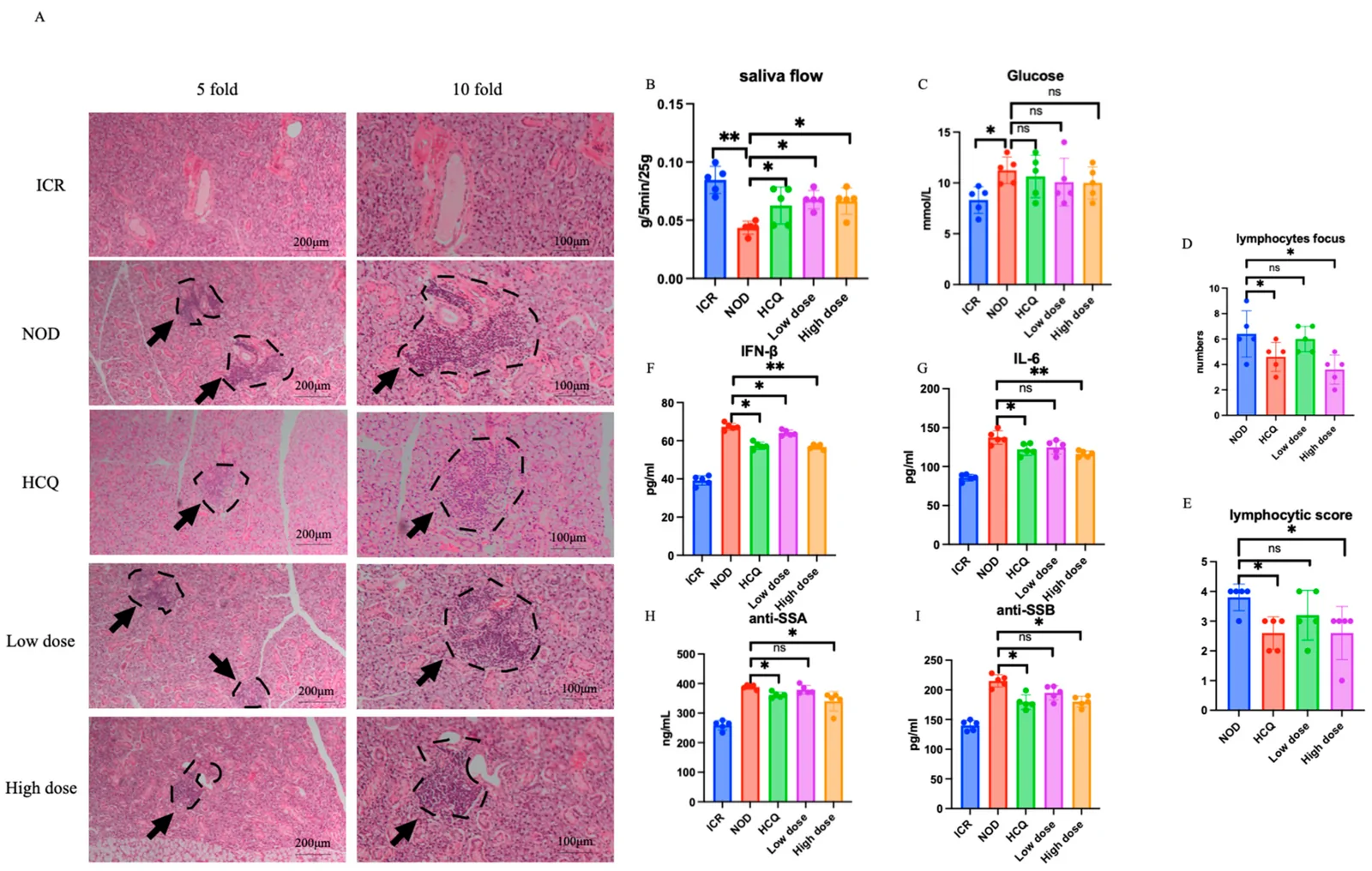
Exploratory pharmacodynamic study of phloretin in animal experiments. (**A**) HE staining indicated that both hydroxychloroquine and phloretin inhibited the formation of lymphocytic infiltration foci. Arrows indicate lymphocytic foci. (**B**) Salivary flow rate measurement showed that both hydroxychloroquine and phloretin improved salivary secretion function. (**C**) Blood glucose detection revealed that hydroxychloroquine and phloretin had no effect on blood glucose levels. (**D**) Statistical analysis of the number of lymphocytic infiltration foci via HE staining demonstrated that the efficacy of phloretin was comparable to hydroxychloroquine. (**E**) Pathological scoring confirmed that phloretin exerted comparable therapeutic effects to hydroxychloroquine. (**F**–**I**) ELISA results of peripheral blood IFN-β, IL-6, anti-SSA and anti-SSB levels indicated that phloretin possessed comparable efficacy relative to hydroxychloroquine. * *p* < 0.05, ** *p* < 0.01; ns, not statistically significant.

As shown in Figure 3, salivary flow rate detection indicated that continuous drug administration effectively alleviated hyposalivation in mice. Blood glucose measurement confirmed that the immunomodulatory effect of phloretin was independent of hypoglycemic side effects (Figure 3 B,C).

HE staining and quantitative pathological analysis of submandibular gland tissues demonstrated that phloretin markedly reduced the number of lymphocytic foci and decreased pathological scores with statistically significant differences (Figure 3A,D,E). The therapeutic efficacy of low-dose phloretin was comparable to that of hydroxychloroquine.

In addition, peripheral blood detection revealed that phloretin inhibited the secretion of interferon-β (IFN-β) and interleukin-6 (IL-6) by dendritic cells and downregulated the levels of autoantibodies including anti-SSA and anti-SSB (Figure 3F–I).

### 2.3. Analysis of Major Immune Cell Subsets in Salivary Gland Infiltration of SS Model Mice

Early studies regarded T and B cells as the dominant cells responsible for the formation of lymphocytic infiltrative foci in Sjögren’s syndrome, due to their high abundance within these lesions. However, limited research has focused on the involvement of upstream innate immune cells. Accumulating evidence [12,13] has highlighted the critical role of innate immunity in the pathological progression of Sjögren’s syndrome. Blocking disease progression by targeting upstream innate immune mechanisms may therefore hold great significance for the early intervention and treatment of this disease.

**Figure 4:**
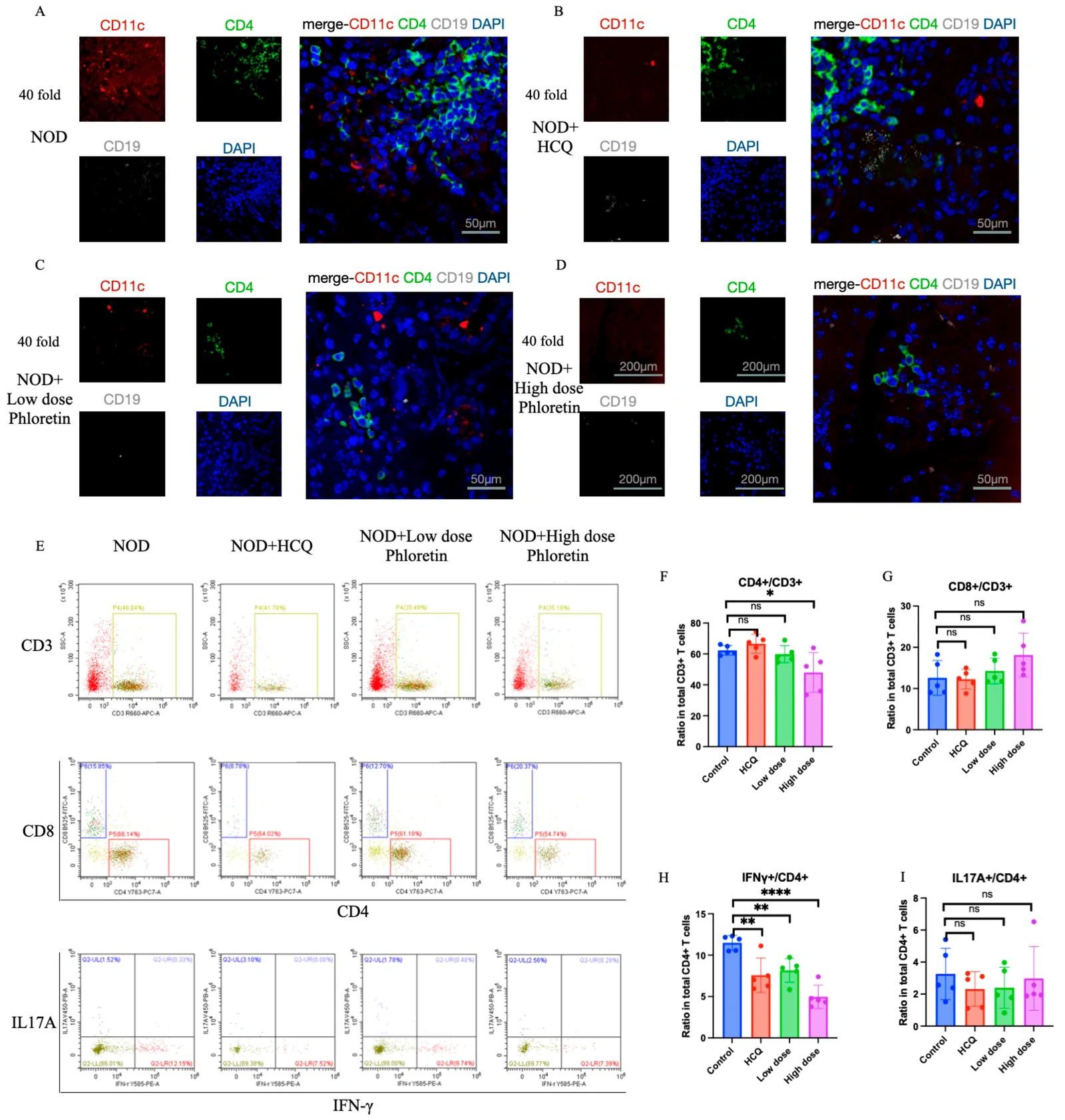
Exploratory pharmacodynamic study of phloretin in animal models (**A**–**D**) Multiplex immunohistochemical analysis revealed the infiltration of CD4/CD19/CD11c-positive cells in each group (magnification: 40×). (**E**) Flow cytometric analysis of immune cells in salivary glands. Green gates in the top row indicate the percentage of CD3^+^ cells. In the middle row, blue gates represent CD8^+^ cells and red gates represent CD4^+^ cells. Quadrant gating in the bottom-row dot-plots shows the frequencies of IFN-γ^+^ and IL-17A^+^ cells among CD4^+^ T cells. (**F**–**I**) Flow cytometry statistics indicated that high-dose phloretin inhibited the infiltration of total CD4^+^ and CD4^+^IFN-γ^+^ cells with statistically significant differences. * *p* < 0.05, ** *p* < 0.01, **** *p* < 0.0001; ns, not statistically significant.

The salivary glands are the primary target organs in Sjögren’s syndrome models. In this study, mouse salivary gland tissues were collected to analyze the infiltration of multiple immune cell populations. Multicolor immunohistochemical staining was performed on mouse submandibular glands to label the dendritic cell marker CD11c, T cell marker CD4 and B cell marker CD19. Combined with HE staining for localization of lymphocytic foci, cell distribution was observed by laser confocal microscopy. Consistent with labial gland tissues from SS patients, CD11c^+^ dendritic cells accumulated around submandibular gland infiltrative foci, which were dominated by CD4^+^ T cells with scattered CD19^+^ B cells. Dendritic cells were confirmed to mediate innate immune activation and lymphocytic lesion formation in SS. As shown in Figure 4, Phloretin notably reduced the infiltration of CD11c^+^ dendritic cells and suppressed excessive local innate immune activation (Figure 4A).

Flow cytometry was used to further analyze glandular lymphocyte subsets. After gating and viable cell screening, phloretin significantly decreased the infiltration of CD4^+^ T cells and IFN-γ^+^ Th1 cells with statistical significance, while no obvious changes were observed in CD8^+^ T cells and IL-17A^+^ Th17 cells among groups. Hydroxychloroquine only reduced Th1 cell infiltration without affecting CD4^+^ and CD8^+^ T cells. In conclusion, phloretin inhibits the salivary gland infiltration of Th1 cells downstream of dendritic cells (Figure 4E–I).

### 2.4. Phloretin Inhibits STING/p-TBK1-Positive Cell Infiltration in Salivary Glands of SS Model Mice

Based on the previous literature review and preliminary drug screening, we hypothesized that phloretin might regulate innate immunity in Sjögren’s syndrome via the STING/TBK1 pathway. In our previous study (Figure 4A–D), we confirmed the infiltration of dendritic cells—the core antigen-presenting cells governing innate immunity in Sjögren’s syndrome, labeled by CD11c—within lymphocytic infiltrative foci in the salivary glands of Sjögren’s syndrome model mice. This pathological manifestation was highly consistent with that observed in human labial gland samples (Figure 1). Moreover, the number of CD11c-positive cells was markedly decreased in the phloretin-treated group compared with the positive control group. Nevertheless, whether phloretin exerts a pharmacological inhibitory effect on the recruitment of CD11c-positive cells into lymphocytic infiltrative foci remains unclear, and the underlying mechanism has not yet been elucidated. In addition, it is still uncertain whether this biological process is mediated by the STING/TBK1 as we speculated. In the current study, we further observed and analyzed the spatial localization of STING/TBK1 and CD11c-positive cells, and presented representative histological images of each pharmacological dose group.

**Figure 5:**
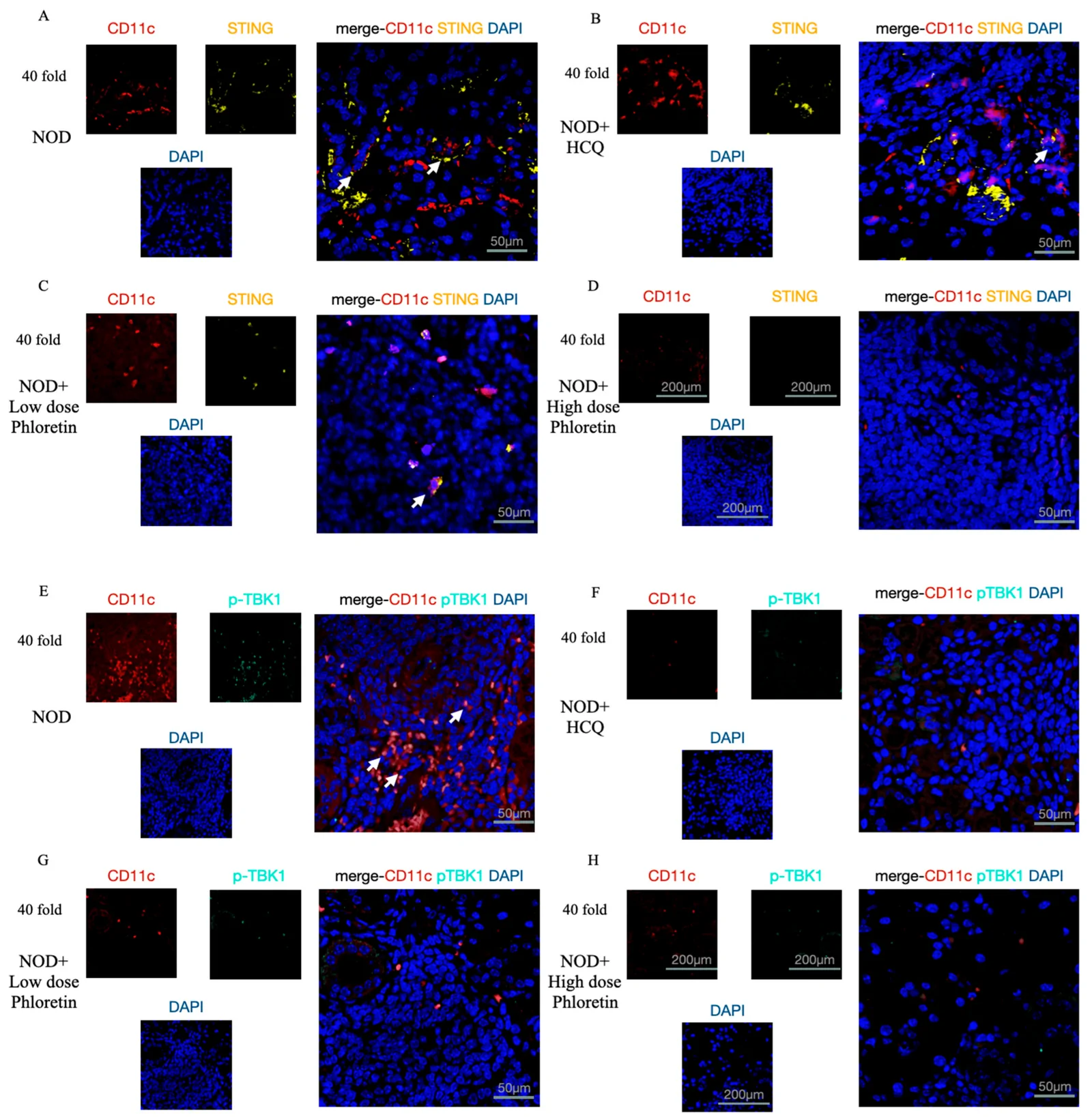
Pharmacodynamic study of phloretin in animal experiments. (**A**–**D**) Multiplex immunohistochemical analysis showed infiltration of CD11c-/STING-positive cells in each group (magnification: 40×); arrows indicate representative co-localized cells. (**E**–**H**) Multiplex immunohistochemical analysis showed infiltration of CD11c/p-TBK1-positive cells in each group (magnification: 40×); arrows indicate representative co-localized cells.

To explore the role of the STING and downstream TBK1 in SS pathogenesis, the expression of p-TBK1 in mouse submandibular glands was further detected. With HE staining for the localization of lymphocytic foci, laser confocal microscopy revealed obvious aggregation of STING^+^ (Figure 5A–D) or p-TBK1^+^ (Figure 5E–H) cells in inflammatory lesions, and STING/p-TBK1 co-localized with CD11c on dendritic cells. Quantitative statistical analysis (Figure S4) was performed to quantify the number of infiltrated CD11c^+^ cells per square millimeter of glandular tissue. Moreover, Manders’ overlap coefficient analysis was applied to evaluate the co-localization level of STING^+^/p-TBK1^+^CD11c^+^ triple-positive cells. Consistent with the results in Figure 5 and Figure S4, these data confirmed that aberrant activation of the STING-pTBK1 signaling pathway drives SS disease progression. Supplementary Figure S4 further demonstrated that phloretin significantly alleviated the infiltration of CD11c^+^ dendritic cells as well as STING^+^/p-TBK1^+^CD11c^+^ triple-positive cells, consequently restraining the overactivated STING/TBK1-dependent innate immune response.

### 2.5. Inhibitory Effect and Mechanism of Phloretin on the Activation, Migration and Maturation of Primary Dendritic Cells

Murine bone marrow monocytes were induced to differentiate into bone marrow-derived dendritic cells (BMDCs) with GM-CSF and IL-4. All cytological experiments in this study were performed using primary BMDCs. Typical dendritic morphology was observed on day 6 of induction [29,30]. Poly I:C was used as an activator for BMDCs to induce the expression of functional co-stimulatory molecules. This study aimed to investigate the mechanisms underlying DC activation, migration and maturation, as well as the regulatory effect of phloretin.

**Figure 6:**
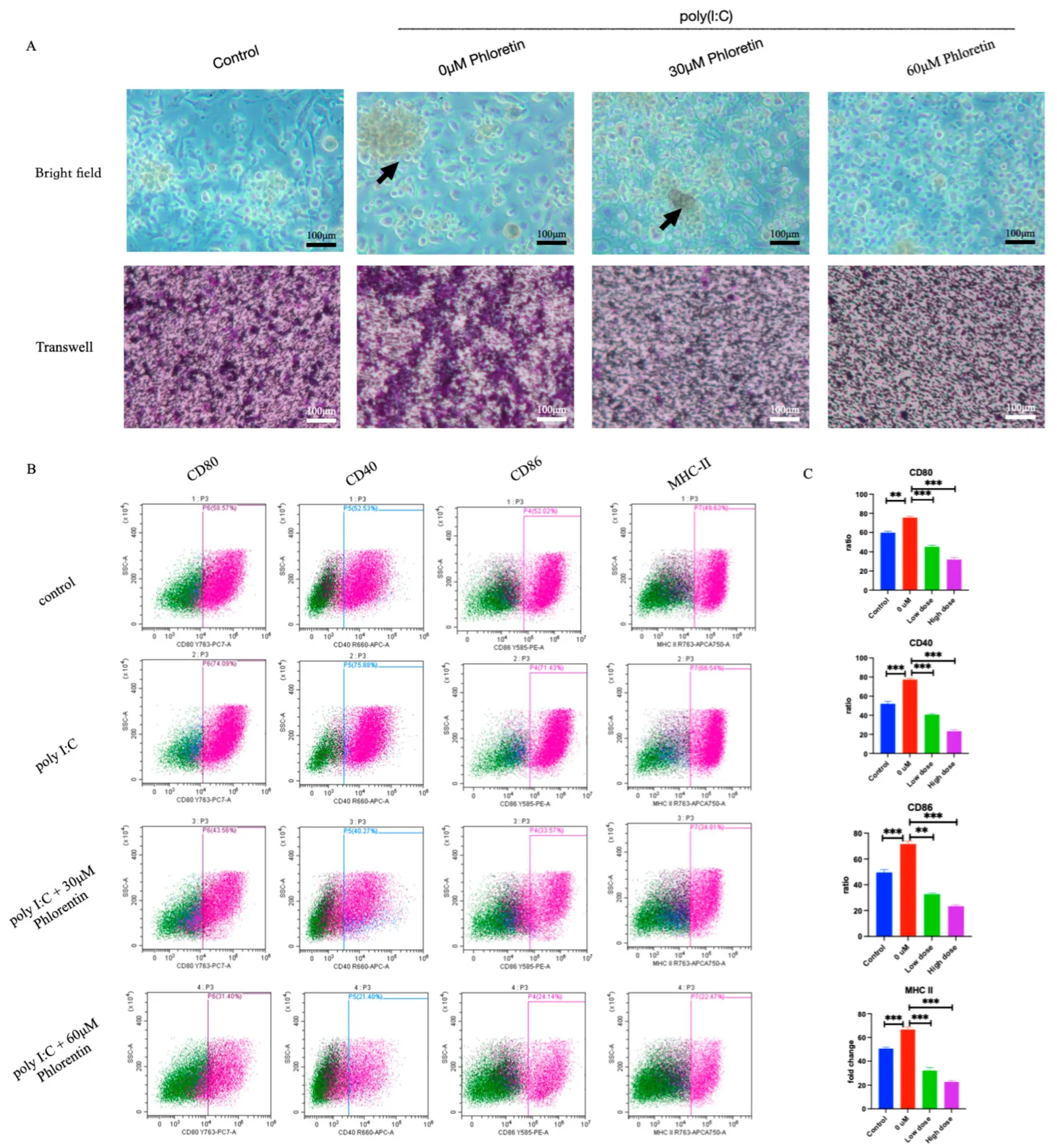
Inhibitory effects and underlying mechanisms of phloretin on the activation, migration and maturation of BMDCs. (**A**) Bright-field microscopy showed cell aggregation, and Transwell assay indicated cell migration. Arrows indicate mature cell clusters. The results suggested that phloretin suppressed the maturation and migration of poly I:C-stimulated BMDCs. (**B**) Flow cytometry detected the expression of CD80, CD40, CD86 and MHC II in BMDCs. (**C**) Statistical analysis of flow cytometry data revealed that phloretin inhibited the expression of surface co-stimulatory molecules in poly I:C-activated BMDCs, with statistically significant differences. ** *p* < 0.01, *** *p* < 0.001.

Four experimental groups were established: blank control, poly I:C-induced model, and low- and high-dose phloretin intervention groups. Microscopic observation showed that poly I:C markedly promoted BMDC aggregation, whereas phloretin reversed this alteration (Figure 6A). Transwell assay and crystal violet staining confirmed that poly I:C enhanced BMDC migration, while phloretin suppressed DC migration in a dose-dependent manner (Figure 6A).

As shown in Figure 6, DCs regulate CD4^+^ T cell immune responses via surface molecules. Flow cytometry demonstrated that poly I:C upregulated the expression of maturation and co-stimulation markers (MHCII, CD40, CD80, CD86) in BMDCs (Figure 6B,C). Phloretin counteracted DC hyperactivation, inhibited DC maturation, and blocked downstream immune signaling.

### 2.6. Phloretin Antagonized Poly I:C-Induced Nuclear Translocation of STAT3 and IRF3 in Primary BMDCs

Based on animal experiments, we found that phloretin reduced the number of CD11c-positive dendritic cells (DCs), a key innate immune marker, within salivary gland lymphocytic infiltrative foci, thereby inhibiting downstream adaptive immunity. In vivo, phloretin also suppressed the secretion of serum IFN-β, IL-6, and the signature autoantibodies SSA/SSB in mice (Figure 3). The transcription factors STAT3 and IRF3 serve as crucial bridges for DCs to secrete cytokines and regulate downstream adaptive immune responses.

**Figure 7:**
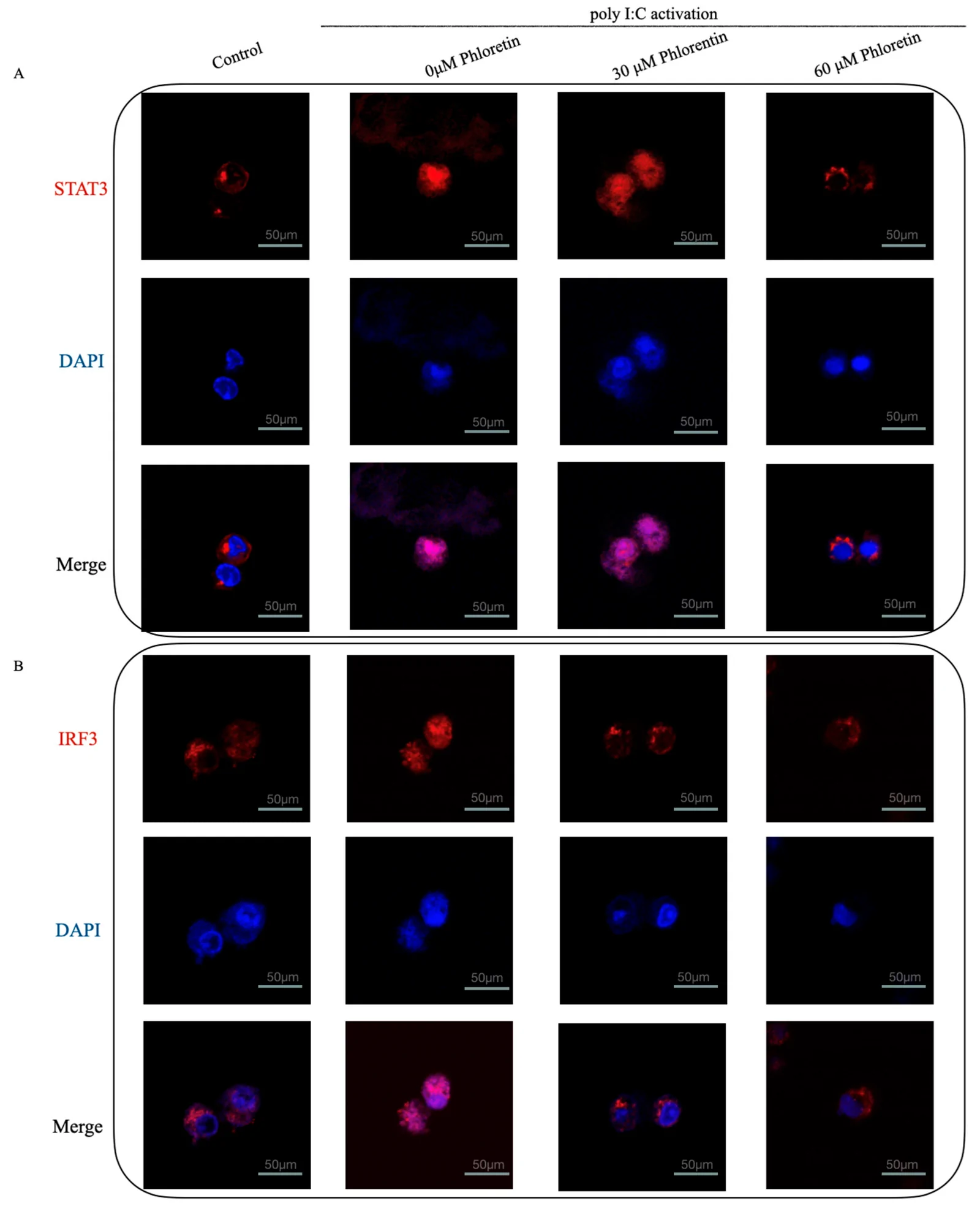
Phloretin blocks nuclear translocation of STAT3 and IRF3 in BMDCs. (**A**) Confocal laser scanning microscopy showed that phloretin inhibited STAT3 nuclear entry in poly I:C-activated BMDCs (magnification: 40×). (**B**) Confocal laser scanning microscopy revealed that phloretin blocked IRF3 nuclear translocation in poly I:C-activated BMDCs (magnification: 40×).

In this section, immunofluorescence results indicate that poly I:C promoted the nuclear translocation and activation of STAT3 and IRF3. Conversely, phloretin significantly blocked this process and inhibited their nuclear entry, as shown in Figure 7.

### 2.7. Phloretin Regulates STING Degradation in Primary BMDCs

Combined with the above phenotypic and cellular observations, we hypothesized that the STING/TBK1/IRF3 innate immune signaling axis is a pivotal driver of aberrant innate immune overactivation in Sjögren’s syndrome (SS). In this work, we systematically dissected the precise molecular mechanism in primary bone marrow-derived dendritic cells (BMDCs), confirming the STING/TBK1/IRF3 cascade serves as the core signaling module governing BMDC-initiated innate inflammatory responses during SS pathogenesis.

**Figure 8:**
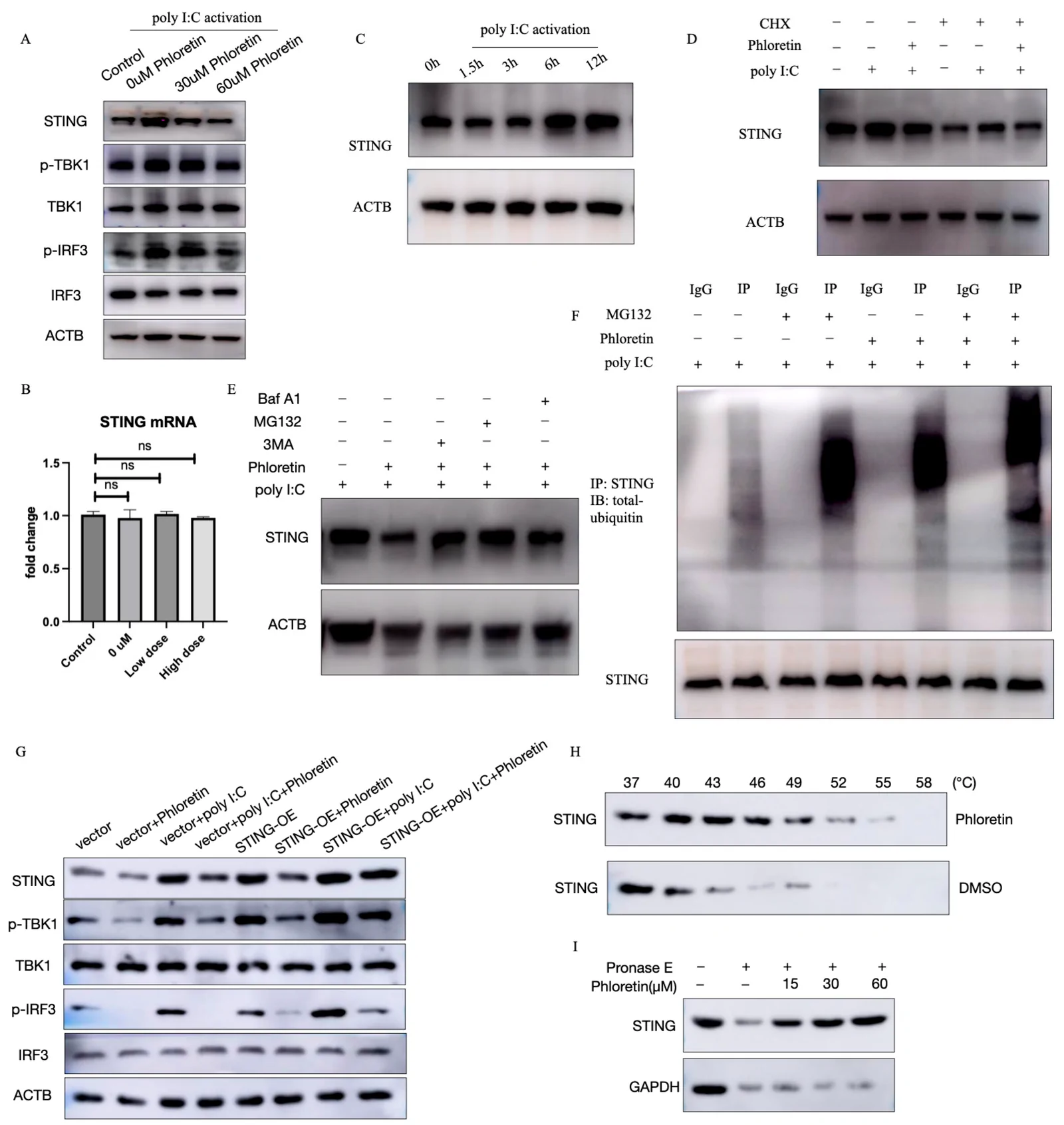
Phloretin interacts with STING to enhance its degradation, leading to the blockade of the STING/TBK1/IRF3 innate immune response triggered by poly I:C in BMDCs. (**A**) Western blot analysis of total STING, phosphorylated TBK1 (p-TBK1), total TBK1, phosphorylated IRF3 (p-IRF3) and total IRF3 protein in BMDCs treated with gradient concentrations of phloretin (0, 30, 60 μM) under poly I:C stimulation; β-actin (ACTB) served as the loading control. (**B**) Relative mRNA expression level of Sting detected by qRT-PCR in phloretin-treated BMDCs with polyI:C stimulation. (**C**) Time-course Western blot detecting STING protein expression at 0 h, 1.5 h, 3 h, 6 h and 12 h post poly I:C treatment. (**D**) Cycloheximide (CHX) chase assay assessing the half-life of endogenous STING protein with or without phloretin and poly I:C co-treatment. (**E**) Western blot detection of STING protein after intervention with autophagy inhibitors (Baf A1, 3MA) and proteasome inhibitor MG132 in the presence of polyI:C and Phloretin. (**F**) Immunoprecipitation-immunoblotting (IP-IB) ubiquitination assay evaluating total ubiquitination modification of STING upon phloretin and MG132 treatment; whole-cell lysate input was shown in the lower panel. (**G**) Rescue Western blot in vector-transfected and STING-overexpressing (STING-OE) cells, verifying STING overexpression abrogates the inhibitory effect of phloretin on p-TBK1 and p-IRF3. (**H**) Cellular thermal shift assay (CETSA) showing the thermal stabilization effect of phloretin on STING protein compared with DMSO control across a temperature gradient (37–58 °C). (**I**) Pronase E limited proteolysis assay confirming the direct binding between phloretin and STING in a dose-dependent manner; GAPDH was used as the internal reference. ns, not statistically significant.

As illustrated in Figure 8, poly I:C stimulation robustly upregulated total STING protein abundance and triggered robust phosphorylation of TBK1 and IRF3, while the total protein levels of TBK1 and IRF3 remained stable (Figure 8A). Treatment with phloretin counteracted poly I:C-mediated pathway activation, markedly reducing STING protein accumulation and dampening the phosphorylation of downstream TBK1 and IRF3 (Figure 8A).

To pinpoint the post-transcriptional regulatory mode underlying phloretin-mediated STING downregulation, we first performed cycloheximide (CHX) chase assays to block de novo protein synthesis. The CHX degradation curve excluded transcriptional and translational inhibition as the responsible mechanism for reduced STING protein (Figure 8D). Consistently, quantitative RT-PCR showed no significant fluctuation in Sting mRNA levels upon phloretin intervention (Figure 8B), collectively demonstrating that phloretin controls STING protein stability via post-translational modification rather than transcriptional repression.

Subsequent pharmacological inhibitor screening clarified the degradation route of STING protein. The proteasome inhibitor MG132 showed a stronger ability to restore phloretin-induced STING downregulation compared with the autophagy inhibitors Bafilomycin A1 and 3-methyladenine (3-MA), which only exerted modest effects. (Figure 8E). Immunoprecipitation-immunoblotting (IP-IB) ubiquitination assays, in which total ubiquitin was detected by immunoblotting, further suggested that phloretin may enhance the ubiquitination modification of STING, potentially facilitating its subsequent degradation (Figure 8F). Rescue Western blot in vector-transfected and STING-overexpressing (STING-OE) cells, verifying STING overexpression abrogates the inhibitory effect of phloretin on p-TBK1 and p-IRF3 (Figure 8G). Cellular thermal shift assay (CETSA) confirmed the direct physical binding between phloretin and endogenous STING protein, as phloretin treatment significantly elevated the thermal stability of STING across a temperature gradient (Figure 8H). Pronase E limited proteolysis assay also verified the direct interaction, showing phloretin protected STING from protease-mediated degradation in a concentration-dependent manner (Figure 8I).

Collectively, these mechanistic assays prove that phloretin accelerates STING degradation, and consequently blocks the overactivated STING/TBK1/IRF3 innate immune signaling cascade in poly I:C-stimulated BMDCs.

## 3. Discussion

Although CD4^+^ T cells have long been regarded as the predominant infiltrating immune cells and key initiators in the labial glands of Sjögren’s syndrome (SS) patients, adaptive immunity is generally upstream-regulated by innate immunity [31,32]. Accumulating evidence [11,12,13] highlights the pivotal role of innate immunity in disease development. Therefore, this study focused on dendritic cells (DCs), the critical antigen-presenting cells of the innate immune system in clinical samples, which constitutes one of the major innovations of our research. Originating from the bone marrow, immature DCs sense damage or pathogenic signals, migrate to sentinel lymph nodes, and then mature to express major histocompatibility complex molecules and co-stimulatory molecules. Mature DCs infiltrate target tissues, secrete type I interferons to shape an inflammatory microenvironment, and present antigens to trigger adaptive immunity, ultimately driving autoreactive T and B cell-mediated autoimmune responses [33,34,35].

Type I interferons (IFN-α/β) are pivotal for initiating innate immunity and amplifying immune cascades in autoimmune disorders. Elevated circulating type I interferon levels are positively correlated with disease activity in multiple autoimmune diseases. Shared interferon signatures across autoimmune conditions indicate common pathogenic mechanisms underlying systemic autoimmunity [36,37]. Clarifying the regulatory mechanisms of abnormal type I interferon production is essential for developing precise targeted therapies against autoimmune diseases.

STING is a vital innate immune sensor for immunogenic nucleic acids, and SS is closely associated with Epstein–Barr virus infection and related persistent functional RNAs. STING activation acts as a pro-inflammatory signal and facilitates the secretion of type I interferons and pro-inflammatory cytokines. Accumulating evidence has demonstrated that aberrant STING expression contributes to the pathogenesis of various autoimmune diseases [14,38,39,40]. Currently, STING-related immune mechanisms and targeted drugs have attracted extensive research attention, and our findings may provide novel evidence for STING research.

By means of literature research [19,20,21,24,25,26] and computational simulation analysis, we found that phloretin, a natural flavonoid compound, may regulate STING activity and overactivated innate immunity in autoimmune diseases and holds therapeutic potential for Sjögren’s syndrome (SS). A series of experiments were further conducted to verify its therapeutic efficacy. Hydroxychloroquine is currently the only first-line drug targeting aberrant innate immune activation for Sjögren’s syndrome clinical treatment, leaving limited therapeutic options. This study aims to expand the research scope of candidate drugs targeting innate immunity of the disease. Clinical practice indicates that hydroxychloroquine presents weaker immunomodulatory effects compared with glucocorticoids. In addition, it barely relieves dry mouth and dry eyes, which severely impair patients’ quality of life. Accordingly, we intend to explore the therapeutic potential of phloretin for better clinical outcomes.

Hydroxychloroquine (HCQ), a first-line agent for SS [27,28,41], is structurally modified from chloroquine (CQ) via the introduction of a hydroxyl group into the N-ethyl side chain of chloroquine. This structural alteration markedly weakens its permeability across the blood–retinal barrier, improves drug safety and reduces the risk of retinal toxicity, making it ideal for long-term maintenance treatment of autoimmune disorders. Though fully synthetic, chloroquine was originally developed based on quinine, a natural alkaloid isolated from the bark of Cinchona trees. As an immunomodulator, HCQ exerts therapeutic effects in autoimmune diseases including systemic lupus erythematosus (SLE), rheumatoid arthritis (RA) and SS by regulating lysosomal function and suppressing excessive innate immune recognition. Its core targets in innate immunity are endosomal pattern recognition receptors, predominantly TLR7 that senses single-stranded RNA and TLR9 that recognizes unmethylated CpG DNA. As a weak lipophilic base, HCQ readily penetrates into lysosomes and endosomes for intracellular accumulation, elevates organelle pH and inhibits the activity of acid-dependent proteases such as cathepsins. It further impairs antigen processing in antigen-presenting cells and blocks the binding of antigenic peptides to MHC class II molecules, thereby restraining the MyD88 signaling cascade and the secretion of pro-inflammatory cytokines, especially type I interferons. Ultimately, it inhibits aberrant activation of autoreactive T cells and maintains immune homeostasis.

This study focuses on phloretin, a natural flavonoid compound. It explores its regulatory effects on poly I:C-induced dendritic cell activation, migration and co-stimulatory molecule expression, as well as the mechanism underlying the STING/TBK1/IRF3 signaling axis associated with STING ubiquitination modification. Future studies can further explore the potential upstream regulatory molecules involved in the phloretin-mediated STING signaling pathway. Existing literature [42,43] has confirmed that multiple E3 ubiquitin ligases and deubiquitinases (DUBs) serve as key regulatory factors for STING activity. Specifically, members of the TRIM and RNF family, including RNF5, TRIM29, and TRIM30a, are capable of suppressing STING activation by facilitating K48-linked ubiquitination and subsequent degradation of STING; meanwhile, various DUBs also participate in the fine-tuning of STING signaling transduction. Nevertheless, whether these STING-related ubiquitination-modified enzymes act as critical upstream regulators in the phloretin-induced STING degradation process remains unclear. Therefore, subsequent research could focus on screening and validating the key E3 ligases and DUBs that mediate the regulatory effect of phloretin on STING, which will help systematically elucidate the upstream molecular mechanism by which phloretin modulates STING signaling.

Phloretin effectively improved salivary secretion function and alleviated excessive innate and adaptive immune responses in NOD/Ltj SS model mice. Despite existing methodological limitations, further investigations on pharmacological mechanism, toxicology and preclinical research will be carried out to elucidate the therapeutic efficacy of phloretin against Sjögren’s syndrome and expand the reservoir of candidate drugs modulating innate immunity for the disease.

Subsequent studies will utilize clinical samples to explore the correlation between STING expression level and disease activity in patients with Sjögren’s syndrome, and further investigate whether STING signaling exhibits disease stage-specific characteristics. The present study only assessed the therapeutic efficacy of phloretin for Sjögren’s syndrome but lacked systematic pharmacokinetic and safety evaluations. Key data regarding blood biochemistry, in vivo drug exposure and bioavailability were not investigated, which limits the clinical translation potential of phloretin. Future work will address these deficiencies by characterizing the in vivo stability, bioavailability, metabolic profile and tissue distribution of phloretin. Comprehensive toxicological analysis and standardized preclinical animal studies will be performed to evaluate its long-term toxicity and metabolic adverse effects, supplement the missing pharmacokinetic and safety evidence, and support its further clinical translation. In addition, the animal experiments in this study adopted a relatively small sample size of five mice per group. Although multiple comparisons were conducted in the statistical analysis, potential statistical bias still remained, which may compromise the reliability of the research results. To mitigate such limitations, increased experimental batches and sample sizes will be adopted in subsequent pharmacokinetic and drug safety evaluations to reduce statistical bias. Furthermore, salivary gland organoids and isolated tissues derived from patients will be applied to replicate and verify the core conclusions of this study, so as to make up for the limitations of animal experiments and further improve the credibility and clinical applicability of the research findings.

## 4. Materials and Methods

### 4.1. Mice

Female NOD/Ltj and ICR mice were purchased from GemPharmatech (Nanjing, China) and housed in a specific pathogen-free (SPF) animal room at a constant temperature and humidity. All animal experiments were performed in accordance with the Declaration of Helsinki to ensure animal welfare. Mice were provided with standard diet and sterile water ad libitum. In terms of animal sample size, we conducted a priori power analysis via G Power 3.1. Referring to published studies on Sjögren’s syndrome animal models, the effect size was set as d = 1.2 (large effect size), with a two-tailed significance level α = 0.05 and statistical power 1 − β = 0.8. A total of 20 female NOD/Ltj mice were randomly divided into four groups ((*n* = 5) per group): (1) NOD/Ltj negative control group; (2) NOD/Ltj + 50 mg/kg hydroxychloroquine (HCQ) group; (3) NOD/Ltj + 25 mg/kg phloretin low-dose group; (4) NOD/Ltj + 75 mg/kg phloretin high-dose group. Dosage of phloretin was set according to previous studies [23,24,25]. Another five female ICR mice were used as healthy negative controls. Starting at 8 weeks of age, mice received intragastric administration of corresponding drugs or vehicle solution for 8 consecutive weeks. Salivary flow rate was determined by intraperitoneal administration of pilocarpine hydrochloride (Sigma-Aldrich, St. Louis, MO, USA) at a dose of 4 mg/kg. Saliva was collected continuously for 5 min starting 5 min after injection, and the obtained saliva volume was normalized to a standard body weight of 25 g. All animals were anesthetized and sacrificed at 16 weeks of age, and tissue samples were collected for subsequent experimental analyses. Blind evaluation was adopted throughout the experimental process to effectively minimize potential observer bias and ensure the objectivity and reliability of experimental results.

### 4.2. Reagents and Antibodies

Phloretin (purity, 98%) was obtained from Macklin Biochemical Co., Ltd. (Shanghai, China). All immunohistochemistry and Western blotting primary and secondary antibodies were purchased from Abcam (Cambridge, UK). All cytokines, flow cytometry (FCM) antibodies, Activation Cocktail (with Brefeldin A), permeabilization buffer, Perm/Wash buffer and ELISA kits were purchased from Dakewe Biotech Co., Ltd. (Shenzhen, China). Other auxiliary experimental reagents were supplied by Beyotime Biotechnology (Shanghai, China).

### 4.3. Hematoxylin–Eosin (HE) Staining

Mouse salivary gland, liver and kidney tissues were isolated and fixed in 4% paraformaldehyde overnight. Tissue embedding and serial sectioning were performed on the next day. Sections were stained using a commercial HE staining kit. After mounting, pathological examination and slice interpretation were completed by a professional pathologist.

### 4.4. Multiplex Immunohistochemistry

Tissue sections were deparaffinized and subjected to antigen retrieval. After permeabilization with 0.1% Triton X-100, sections were blocked with 10% goat serum. Following gentle washing with PBST, sections were incubated with primary antibodies overnight at 4 °C, followed by one-hour incubation with secondary antibodies. After PBST washing, sections were incubated with TSA signal amplification solution in the dark. Nuclei were counterstained with DAPI, and anti-fade mounting medium was added. Images were captured by laser confocal scanning microscopy.

### 4.5. Enzyme-Linked Immunosorbent Assay (ELISA)

Sample coating, loading, incubation and color development were conducted strictly in accordance with the manufacturer’s instructions of ELISA kits. The standard curve was plotted to calculate the target protein concentration in each sample.

### 4.6. Primary BMDC (Bone Marrow-Derived Dendritic Cells) Culture

Under sterile conditions, femurs and tibias were isolated from female C57BL/6 mice, and surrounding muscle tissues were completely removed. Mouse bones were disinfected with 75% ethanol, and bone marrow cells were flushed out with a sterile syringe. Cell suspensions were filtered through a 70 μm cell strainer, and viable cells were counted using the trypan blue exclusion method. Bone marrow-derived dendritic cells (BMDCs) were induced by 20 ng/mL GM-CSF plus 10 ng/mL IL-4 over 7 days with half medium changes every two days. Flow cytometry was used to verify cellular purity for critical batches at day 7 harvest.

### 4.7. Flow Cytometry Staining of Animal Tissues

Tissues were minced and digested with type II collagenase solution. Cell viability was detected during digestion to prevent excessive digestion. After digestion, cells were washed and filtered through a 200-mesh sieve placed on a 50 mL centrifuge tube. The collected cell suspension was centrifuged at 400 g for 5 min. Cell pellets were gently resuspended and washed with PBS, followed by centrifugation at 400 g. Cells were resuspended in serum-supplemented RPMI 1640 complete medium, and incubated with appropriate amount of Cell Activation Cocktail (with Brefeldin A) for 6 h according to cell quantity. After PBS washing and centrifugation at 400 g for 5 min, cells were stained with Zombie dye for 15 min to label dead cells. After another PBS washing, cells were blocked with Fc blocker. Subsequently, cells were incubated with surface marker antibodies on ice in dark for 15 min as instructed by the manufacturer. After washing, cells were fixed with 4% paraformaldehyde for 15 min, and permeabilized with permeabilization buffer for 15 min. Cells were rinsed gently with Perm/Wash buffer after permeabilization. Intracellular cytokine antibodies diluted with Perm/Wash buffer were added and incubated on ice in dark for 15 min. Finally, cells were washed and resuspended for flow cytometry analysis.

### 4.8. Western Blotting

Collected samples were fully lysed in RIPA lysis buffer supplemented with protease and phosphatase inhibitors. Protein concentration was quantified via the BCA method, and protein samples were denatured by heating with SDS loading buffer. A total of 15–25 μg protein per lane was separated by SDS-PAGE and transferred onto PVDF membranes. Membranes were blocked with 5% skimmed milk at room temperature for 1.5 h, incubated with primary antibodies overnight at 4 °C, and then hybridized with secondary antibodies for 1.5 h. Protein bands were visualized using an ECL chemiluminescence system.

### 4.9. Immunofluorescence Staining

Cell samples were fixed and permeabilized with 0.1% Triton X-100, followed by blocking with 10% goat serum. After PBST washing and gentle centrifugation, cell precipitates were incubated with primary antibodies overnight and secondary antibodies for 1 h. Nuclei were stained with DAPI after PBST rinsing. Finally, anti-fade reagent was added, and images were acquired using a laser confocal microscope.

### 4.10. Reverse Transcription Quantitative PCR (RT-qPCR)

Total RNA was extracted from cells using the TRIzol reagent. RNA concentration and purity were determined, and qualified RNA was reverse-transcribed into complementary DNA (cDNA). The relative mRNA expression levels of target genes were detected by real-time quantitative PCR.

### 4.11. Immunoprecipitation (IP)

Collect cell samples, lyse the cells and perform ultrasonication, then centrifuge to collect the supernatant. Immunoprecipitation (IP) was carried out using STING-specific antibodies to enrich all intracellular STING proteins, including unubiquitinated and polyubiquitinated modified STING. Samples were pre-incubated with continuous rotation at 4 °C overnight. After three thorough washes, magnetic beads were co-incubated with protein samples at 4 °C for 3 h. Finally, Western blot assay was performed with ubiquitin (Ub) antibody to detect the ubiquitination modification bands of STING protein in the immunoprecipitation products.

### 4.12. Molecular Docking and Molecular Dynamics Simulation

The 3D crystal structure of STING protein was downloaded from the PDB database, and the structure of phloretin ligand was obtained from the PubChem database. Both protein and small-molecule structures were preprocessed uniformly, including removal of water molecules and redundant heteroatoms, addition of polar hydrogens, as well as geometric optimization and energy minimization. The potential active binding pocket of STING was predicted to define the docking grid box, and molecular docking simulation was executed with AutoDock software v1.1.2 to screen the optimal binding pose and calculate the binding free energy between phloretin and STING. Subsequently, the top-ranked docking complex with the lowest binding energy was subjected to molecular dynamics (MD) simulation using GROMACS software v2026.1. The system was subjected to energy minimization, NVT constant-volume temperature equilibration and NPT constant-pressure density equilibration stepwise, followed by formal productive dynamics simulation. Root-mean-square deviation (RMSD), root-mean-square fluctuation (RMSF), hydrogen bond occupancy and binding free energy decomposition were analyzed to evaluate the binding stability, dynamic conformational changes and sustained interaction strength of phloretin within the STING binding pocket.

### 4.13. STING Overexpression Rescue Assay

DC2.4 cells were seeded into 24-well plates and transiently transfected with STING overexpression plasmid or empty vector control via Lipofectamine 3000 (Thermo Fisher Scientific, Waltham, MA, USA) following the manufacturer’s instructions. Six hours after transfection, the transfection medium was replaced with fresh complete RPMI-1640 medium to reduce liposome-mediated cytotoxicity and non-specific activation of innate immune signaling. At 24 h post-transfection, cells were subjected to subsequent treatments, including phloretin incubation and polyI:C stimulation, to perform rescue functional experiments. Finally, cells were lysed for Western blot detection of STING, phosphorylated TBK1, total TBK1, phosphorylated IRF3 and total IRF3 protein levels, aiming to verify whether STING overexpression could reverse the inhibitory effect of phloretin on the STING-TBK1-IRF3 signaling cascade.

### 4.14. CETSA and DARTS

Cellular Thermal Shift Assay (CETSA) was used to detect the intracellular direct binding between phloretin and STING. Cells were treated with equivalent volumes of DMSO or phloretin, harvested and lysed to prepare total protein lysates. Protein samples from both groups were heated at a serial temperature gradient of 37 °C, 40 °C, 43 °C, 46 °C, 49 °C, 52 °C, 55 °C and 58 °C for thermal denaturation. After freeze–thaw cycles, insoluble heat-aggregated proteins were removed by centrifugation, and soluble supernatants were subjected to Western blot for detection of retained STING protein. Drug Affinity Responsive Target Stability (DARTS) assay was performed to verify the in vitro physical interaction between phloretin and STING. Total cell lysates were divided into five equal groups: group 1 was undigested control without Pronase E and phloretin; group 2 was digested with Pronase E alone; groups 3 to 5 were pre-incubated with 15 μM, 30 μM and 60 μM phloretin at room temperature prior to limited proteolysis by Pronase E. The reaction was terminated, and residual STING protein was examined by Western blotting with GAPDH as the internal loading control.

### 4.15. Ethics

Clinical research was conducted in accordance with the principles of the Declaration of Helsinki. The study protocol was approved by the Hospital Ethics Committee (Approval No. SH9H-2019-T159-2). Written informed consent was obtained from all participants. Before signing the consent forms, all participants were fully informed regarding anonymity, the purpose of the study, the usage of data, and any potential risks associated with participation. All animal experiments were strictly performed in accordance with the principles of the Declaration of Helsinki and the ARRIVE guidelines, fully complying with standard animal welfare regulations and relevant ethical norms. All experimental protocols were reviewed and formally approved by the Animal Experiment Center & Hospital Ethics Committee (Approval No. SH9H-2023-A603-1).

### 4.16. Statistical Analysis

All experiments were repeated at least three times. Data analysis was performed using GraphPad Prism 9.0 and SPSS 19.0 software. All numerical results were presented as mean ± standard deviation (SD). Normality was tested using the Shapiro–Wilk test, and homogeneity of variance was evaluated prior to group comparisons. Normally distributed data with equal variance were analyzed by one-way ANOVA followed by Tukey’s post hoc test for multiple pairwise comparisons. Non-normal datasets were subjected to the non-parametric Kruskal–Wallis test with Dunn’s post hoc correction. Bonferroni adjustment for cross-endpoint global multiple testing correction. *p* value < 0.05 was considered statistically significant (* *p* < 0.05, ** *p* < 0.01, *** *p* < 0.001). NS denotes no statistically significant difference between groups.

## 5. Conclusions

In vivo and primary cell experiments confirmed that CD11c^+^ DCs and STING-mediated innate immunity are closely involved in SS progression, especially the formation of salivary gland lymphocytic foci. Phloretin effectively improved salivary secretion function and alleviated excessive innate and adaptive immune responses in NOD/Ltj SS model mice. In vitro, phloretin inhibited poly I:C-induced DC activation, migration and co-stimulation molecule expression, and regulated the STING/TBK1/IRF3 axis associated with STING ubiquitination modification. Collectively, this study validated that phloretin is a promising candidate agent for regulating DC dysfunction in SS at the animal, cellular and molecular levels.

## Data Availability

The original contributions presented in this study are included in the article/Supplementary Material. Further inquiries can be directed to the corresponding author(s).

## Supplementary Materials

The following supporting information can be downloaded at: https://www.mdpi.com/article/10.3390/ph19091398/s1, Figure S1: STING mRNA expression levels in peripheral blood mononuclear cells (PBMCs) and minor salivary glands (MSGs) of Sjögren’s syndrome (SS) patients across multiple GEO datasets. (A) Box-and-whisker scatter plot showing VST+RSN normalized STING expression in PBMCs from healthy controls and SS patients in GSE66795. No statistically significant difference was observed between groups (ns, not significant). (B) Batch-corrected RMA log2-transformed STING expression in minor salivary glands stratified by disease progression stages (Control, Early, Moderate, Advanced SS) in GSE23117. Significant upregulation was detected in Advanced SS compared with controls (* *p* < 0.05), while comparisons between Control vs. Early and Control vs. Moderate showed no significance (ns). Black dots = Batch 1 samples; triangles = Batch 2 samples. (C) Batch-corrected VST-normalized STING mRNA levels in minor salivary glands from healthy individuals and SS patients with TLS-associated lesions in GSE272410. STING was markedly elevated in TLS-positive gland lesions (**** *p* < 0.0001). (D) Gradient upregulation of batch-corrected VST-normalized STING expression in minor salivary glands according to the severity of focal lymphocytic infiltrates (Healthy, Small foci, Large foci, GC = germinal center-containing lesions) in GSE272410. Statistical significance: ** *p* < 0.01, **** *p* < 0.0001. All boxplots display median, interquartile range, and individual sample scatter points. ns, non-significant.; Figure S2: Phloretin exerts no toxic effects on the liver and kidneys. (A) No obvious pathological abnormalities were observed in liver tissues of each group by hematoxylin-eosin (HE) staining. (B) No obvious pathological abnormalities were observed in kidney tissues of each group by hematoxylin-eosin (HE) staining; Figure S3: Gating strategy for flow cytometric detection of Th1 and Th17 cells in peripheral blood mononuclear cells (PBMCs) with fluorescence minus one (FMO) controls. First gate P1: Total intact leukocytes were preliminarily delineated according to forward scatter area (FSC-A) and side scatter area (SSC-A);Second gate P3: CD45^+^ immune cells were further screened from the P1 parent population; Third gate P4: CD3^+^ T lymphocytes were gated within CD45^+^ cells; Fourth gate P5: CD3^+^CD4^+^ helper T cells were isolated from total CD3^+^ T cells, with CD8^+^ T cells defined as P6; Final dot plot: CD4^+^ T cells were subdivided into four quadrants based on intracellular IFN-γ and IL-17A expression: Q2-UR for IFN-γ^+^IL-17A^+^ double-positive cells, Q2-UL for IFN-γ single-positive Th1 cells, Q2-LR for IL-17A single-positive Th17 cells, and Q2-LL for double-negative cells. All threshold settings and negative boundaries were strictly calibrated using fluorescence minus one (FMO) controls to eliminate non-specific fluorescence interference and ensure accurate positive cell discrimination. The percentage of each gated population is labeled on the corresponding region; Figure S4: Quantitative quantification of CD11c^+^ dendritic cell infiltration and Manders’ co-localization coefficients of STING/phosphorylated TBK1 with CD11c in submandibular glands of NOD mice after different interventions. (A) Statistical density of CD11c^+^ dendritic cells per square millimeter calculated from immunofluorescence images of mouse submandibular gland tissues in four groups: untreated NOD model group, HCQ positive control group, low-dose phloretin group and high-dose phloretin group. (B) Manders’ overlap coefficient reflecting the co-localization degree of STING protein with CD11c^+^ dendritic cells, quantified from immunofluorescence pictures Figure 5A–D. (C) Manders’ overlap coefficient presenting the co-localization level of phosphorylated TBK1 (p-TBK1) with CD11c^+^ dendritic cells, quantified from immunofluorescence pictures Figure 5E–H. All data are expressed as mean ± standard deviation. ns, no significant difference; * *p* < 0.05, ** *p* < 0.01, *** *p* < 0.001; Figure S5: Quantitative gray-scale statistical analysis of Western blot and immunoprecipitation assays exploring the mechanism by which phloretin regulates STING ubiquitination and STING/TBK1/IRF3 signaling cascade. (A) Cells were treated with 0 μM, low-dose and high-dose phloretin under poly I:C stimulation. Relative protein level of total STING in Figure 8A. (B) Phosphorylation ratio of TBK1 (pTBK1/TBK1) in Figure 8A. (C) Phosphorylation ratio of IRF3 (pIRF3/IRF3) in Figure 8A. (D) Gray value quantification of STING protein from Figure 8E Western blot. Autophagy inhibitor 3MA, proteasome inhibitor MG132 and lysosomal inhibitor Bafilomycin A1 (Baf A1) were applied to explore the degradation pathway of STING affected by phloretin. (E) Gray value quantification of total ubiquitination level from immunoprecipitation IP assay in Figure 8F. Groups included poly I:C alone, poly I:C + MG132, poly I:C + phloretin, and poly I:C + phloretin + MG132. All quantitative data are presented as mean ± SD. ns, not statistically significant; * *p* < 0.05, ** *p* < 0.01, *** *p* < 0.001.

## Abbreviations

SS, Sjögren’s syndrome; DC, Dendritic cells; BMDC, bone marrow dendritic cells; HCQ, hydroxychloroquine; IFN, interferons; 3MA, 3-Methyladenine;Baf A1, Bafilomycin A1; CHX, cycloheximide; ub, ubiquitin.
