# Supplementary material for: Phloretin Modulates STING Ubiquitination Degradation to Restrain Dendritic Cell Overactivation and Alleviate Sjögren’s Syndrome
Source: Pharmaceuticals (Basel). 2026 Sep 4;19(9):1398. doi: 10.3390/ph19091398 (PMC13610008; doi:10.3390/ph19091398)
Supplement: Supplementary file 1 [file pharmaceuticals-19-01398-s001.zip › pharmaceuticals-4397924-supplementary.pdf]

pharmaceuticals

MDPI

Supplementary Materials

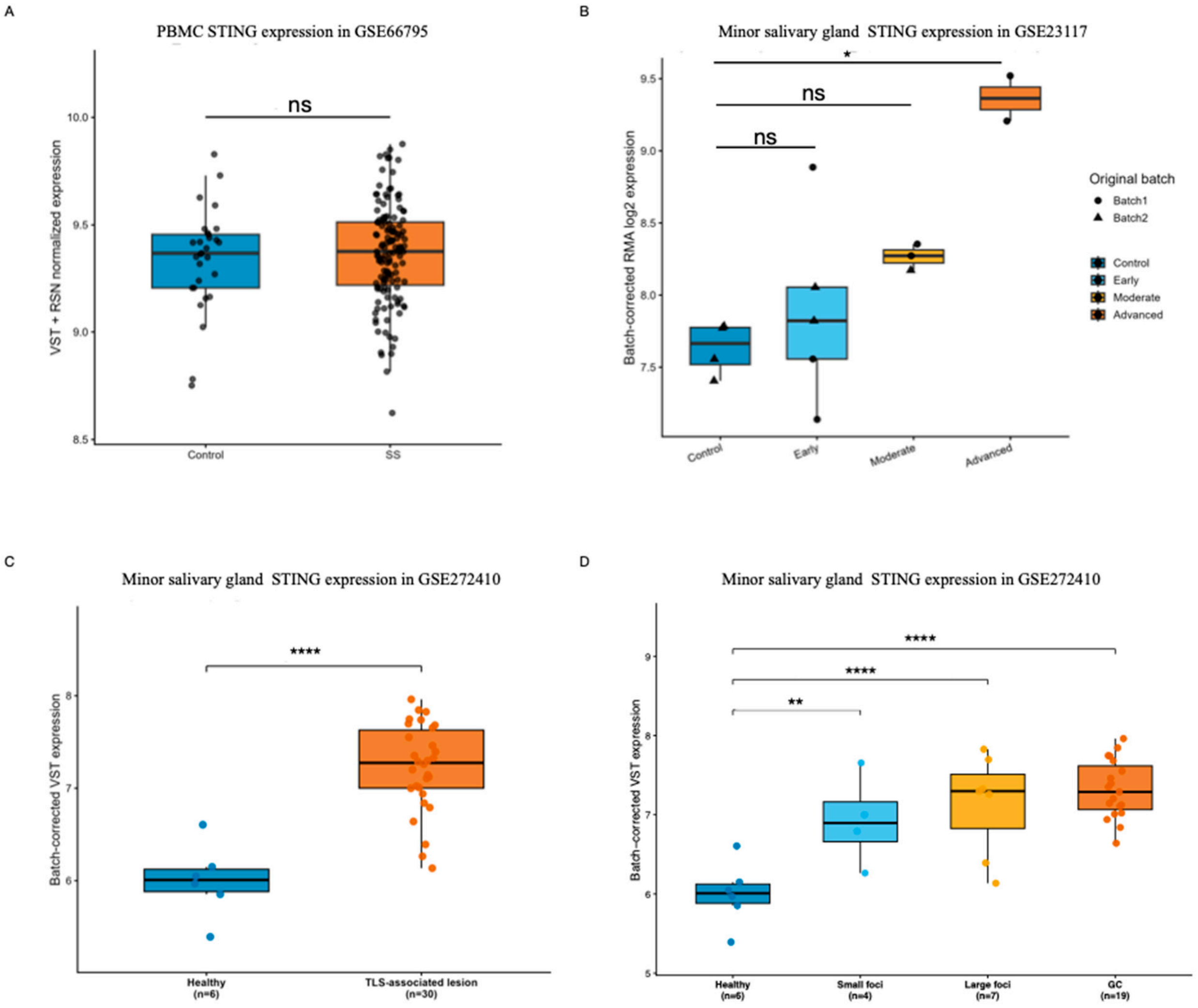

Figure S1. STING mRNA expression levels in peripheral blood mononuclear cells (PBMCs) and minor salivary glands (MSGs) of Sjögren's syndrome (SS) patients across multiple GEO datasets. (A) Box-and-whisker scatter plot showing VST+RSN normalized STING expression in PBMCs from healthy controls and SS patients in GSE66795. No statistically significant difference was observed between groups (ns, not significant). (B) Batch-corrected RMA log2-transformed STING expression in minor salivary glands stratified by disease progression stages (Control, Early, Moderate,

Advanced SS) in GSE23117. Significant upregulation was detected in Advanced SS compared with controls (*P < 0.05), while comparisons between Control vs. Early and Control vs. Moderate showed no significance (ns). Black dots = Batch 1 samples; triangles = Batch 2 samples. (C) Batch-corrected VST-normalized STING mRNA levels in minor salivary glands from healthy individuals and SS patients with TLS-associated lesions in GSE272410. STING was markedly elevated in TLS-positive gland lesions (****P < 0.0001). (D) Gradient upregulation of batch-corrected VST-normalized STING expression in minor salivary glands according to the severity of focal lymphocytic infiltrates (Healthy, Small foci, Large foci, GC = germinal center-containing lesions) in GSE272410. Statistical significance: **P < 0.01, ****P < 0.0001. All boxplots display median, interquartile range, and individual sample scatter points. ns, non-significant.

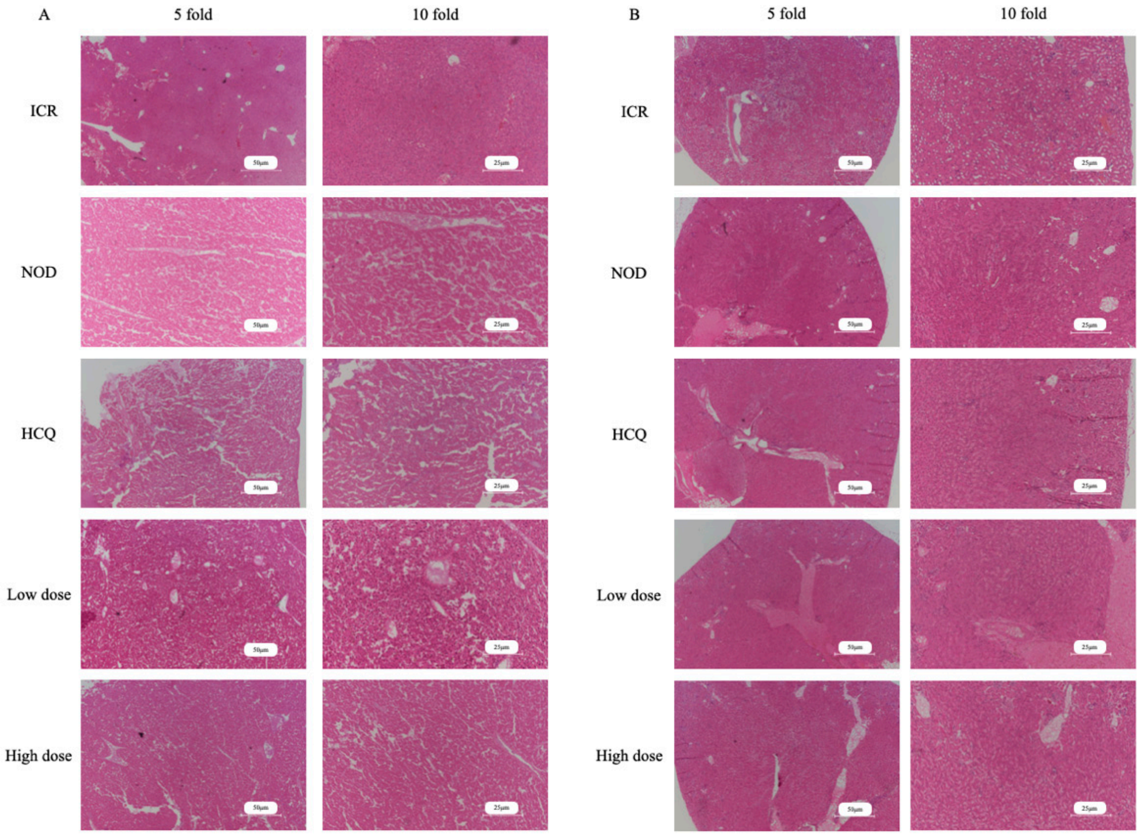

Figure S2. Phloretin exerts no toxic effects on the liver and kidneys. (A) No obvious pathological abnormalities were observed in liver tissues of each group by hematoxylin-eosin (HE) staining. (B) No obvious pathological abnormalities were observed in kidney tissues of each group by hematoxylin-eosin (HE) staining.

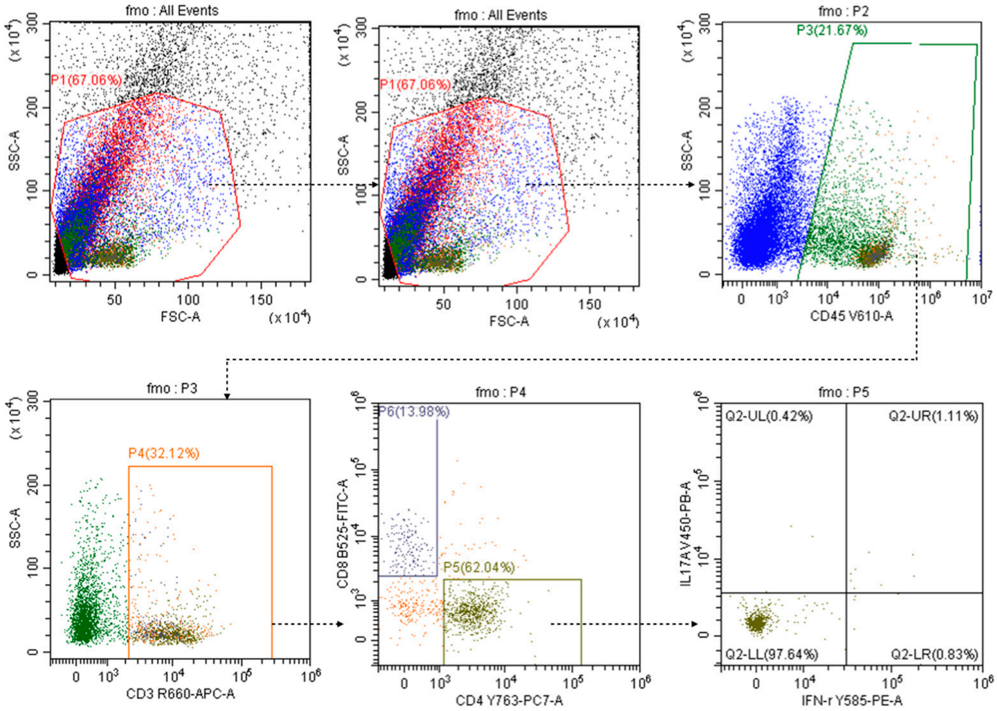

Figure S3. Gating strategy for flow cytometric detection of Th1 and Th17 cells in peripheral blood mononuclear cells (PBMCs) with fluorescence minus one (FMO) controls. First gate P1: Total intact leukocytes were preliminarily delineated according to forward scatter area (FSC-A) and side scatter area (SSC-A);Second gate P3: $CD45^+$ immune cells were further screened from the P1 parent population; Third gate P4: $CD3^+$ T lymphocytes were gated within $CD45^+$ cells; Fourth gate P5: $CD3^+CD4^+$ helper T cells were isolated from total $CD3^+$ T cells, with $CD8^+$ T cells defined as P6; Final dot plot: $CD4^+$ T cells were subdivided into four quadrants based on intracellular IFN-γ and IL-17A expression: Q2-UR for IFN-γ^+IL-17A$^+$ double-positive cells, Q2-UL for IFN-γ single-positive Th1 cells, Q2-LR for IL-17A single-positive Th17 cells, and Q2-LL for double-negative cells. All threshold settings and negative boundaries were strictly calibrated using fluorescence minus one (FMO) controls to eliminate non-specific fluorescence interference and ensure accurate positive cell discrimination. The percentage of each gated population is labeled on the corresponding region.

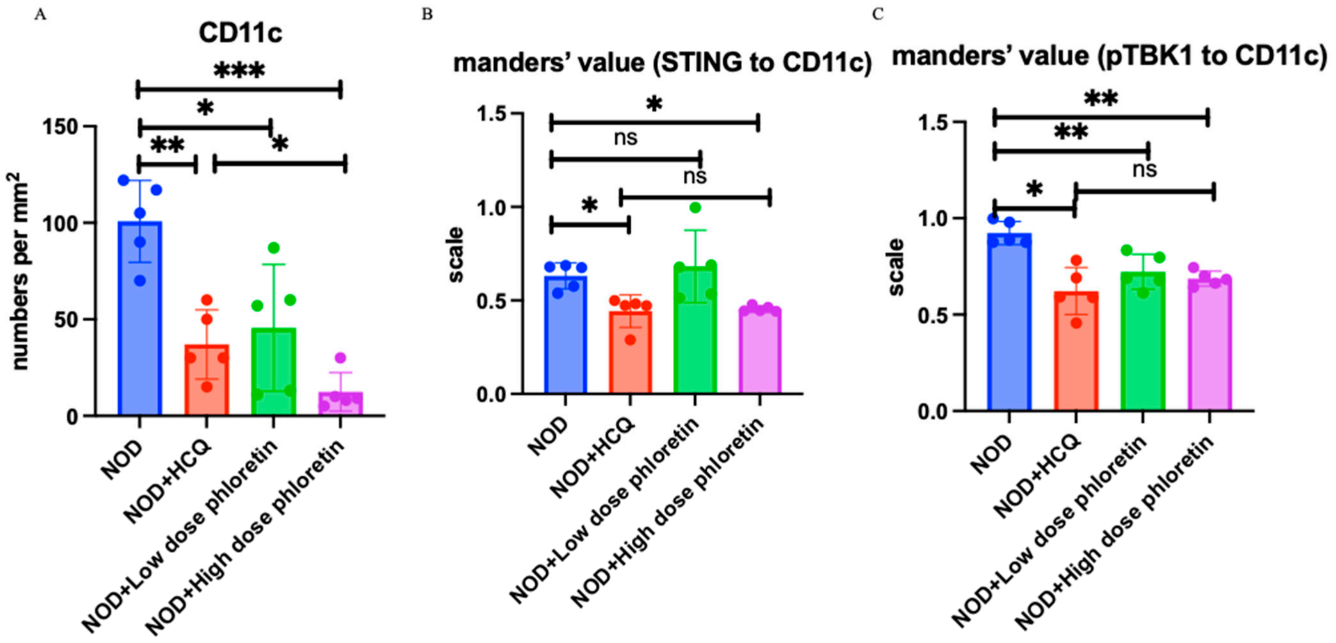

Figure S4. Quantitative quantification of CD11c$^+$ dendritic cell infiltration and Manders' co-localization coefficients of STING/phosphorylated TBK1 with CD11c in submandibular glands of NOD mice after different interventions. (A) Statistical density of CD11c$^+$ dendritic cells per square millimeter calculated from immunofluorescence images of mouse submandibular gland tissues in four groups: untreated NOD model group, HCQ positive control group, low-dose phloretin group and high-dose phloretin group. (B) Manders' overlap coefficient reflecting the co-localization degree of STING protein with CD11c$^+$ dendritic cells, quantified from immunofluorescence pictures Figure 5A–D. (C) Manders' overlap coefficient presenting the co-localization level of phosphorylated TBK1 (p-TBK1) with CD11c$^+$ dendritic cells, quantified from immunofluorescence pictures Figure 5E–H. All data are expressed as mean ± standard deviation. ns, no significant difference; $^*P < 0.05$, $^{**}P < 0.01$, $^{***}P < 0.001$.

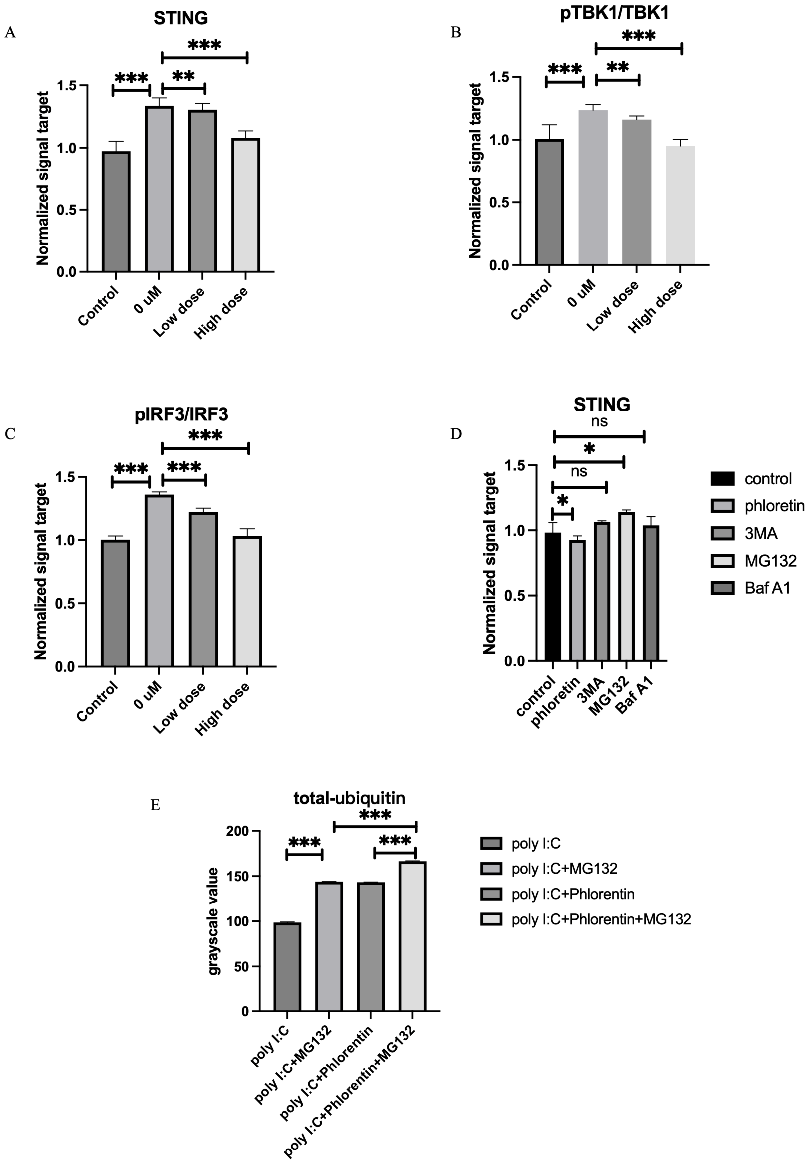

Figure S5. Quantitative gray-scale statistical analysis of Western blot and immunoprecipitation assays exploring the mechanism by which phloretin regulates STING ubiquitination and STING/TBK1/IRF3 signaling cascade. (A) Cells were treated with 0 μM, low-dose and high-dose phloretin under poly I:C stimulation. Relative protein level of total STING in Figure 8A. (B) Phosphorylation ratio of TBK1 (pTBK1/TBK1) in Figure 8A. (C) Phosphorylation ratio of IRF3 (pIRF3/IRF3) in Figure 8A. (D) Gray value quantification of STING protein from Figure 8E Western blot. Autophagy inhibitor 3MA, proteasome inhibitor MG132 and lysosomal inhibitor Bafilomycin A1 (Baf A1) were applied to explore the degradation pathway of STING affected by phloretin. (E) Gray value quantification of total ubiquitination level from immunoprecipitation IP assay in Figure 8F. Groups included poly I:C alone, poly I:C + MG132, poly I:C + phloretin, and poly I:C + phloretin + MG132. All quantitative data are presented as mean ± SD. ns, not statistically significant; *P < 0.05, **P < 0.01, ***P < 0.001.
